# Pure Laparoscopic vs. Open Right Hepatectomy in Living Liver Donors: Bench-Surgery Time

**DOI:** 10.3389/fsurg.2021.771026

**Published:** 2021-11-23

**Authors:** Kwangpyo Hong, Suk Kyun Hong, Eui Soo Han, Sanggyun Suh, Su young Hong, Jeong-Moo Lee, YoungRok Choi, Nam-Joon Yi, Kwang-Woong Lee, Kyung-Suk Suh

**Affiliations:** ^1^Department of Surgery, Seoul National University College of Medicine, Seoul, South Korea; ^2^Department of Surgery, Uijeongbu Eulji Medical Center, Uijeongbu, South Korea

**Keywords:** liver transplantation, living donor, hepatectomy, laparoscopy, bench-surgery

## Abstract

**Background:** Recently, there have been several reports on pure laparoscopic donor right hepatectomy (PLDRH), but the effect of pure laparoscopy on bench surgery has not been evaluated. This study aimed to compare bench-surgery time between PLDRH and conventional donor right hepatectomy (CDRH).

**Methods:** We retrospectively reviewed the medical records of 758 live liver donors between January 2012 and December 2019. We divided the patients into two groups: between January 2012 and September 2015, when we exclusively performed CDRH, and between March 2016 and December 2019, when PLDRH was standardized. We excluded all other types of graft donor hepatectomy, laparoscopic assisted donor hepatectomy, and cases with no recorded data.

**Results:** In total, 267 donors were included in the PLDRH group and were compared with 247 donors in the CDRH group. Similar proportions of graft vascular variations were observed between the two groups. The mean bench-surgery time was longer in the PLDRH group than in the CDRH group (49.3 ± 19.9 vs. 39.5 ± 17.5 min; *P* < 0.001).

**Conclusion:** The bench-surgery time was longer in the PLDRH group than the CDRH group, regardless of whether the vascular network was reconstructed. Expertise in bench-surgery as well as donor surgery and recipient surgery is mandatory for PLDRH to be safe and feasible.

## Introduction

Liver transplantation (LT) is considered the optimal treatment for patients with end-stage liver disease and is a curative treatment for hepatocellular carcinoma. Living donor LT (LDLT) has been implemented as an alternative in countries with a shortage of deceased donors, particularly in Asian countries (1). The first pure laparoscopic donor left lateral sectionectomy was reported in 2002 (2), and subsequently minimally invasive donor hepatectomy (MIDH) was developed.

There have been several studies on pure laparoscopic donor hepatectomy (PLDH) at specialized medical institutions (3–5). In particular, pure laparoscopic donor right hepatectomy (PLDRH) has shown no significant differences in donor safety and feasibility, as well as overall and graft survival of recipients, compared to conventional donor right hepatectomy (CDRH) (6–8). Recently, MIDH, particularly involving pure laparoscopic techniques, is now accepted by international expert consensus guidelines. Considering certain indications, it could become a standard procedure in the future (9). PLDH has been reported to be safe for donors, and its advantages include reduced blood loss, reduced length of hospital stay, and wound satisfaction (4, 5, 7, 10). There are no significant differences reported in recipient outcomes; however, further research is required on long-term outcomes of the recipients, including bile duct complications after LT (7).

Graft quality has a crucial effect on the outcome of recipients in LDLT; however, to the best of our knowledge, bench-surgery time in PLDRH compared with CDRH have not been described yet. Therefore, this study aimed to compare bench-surgery time between PLDRH and CDRH.

## Materials and Methods

### Patients and Methods

**Figure 1:**
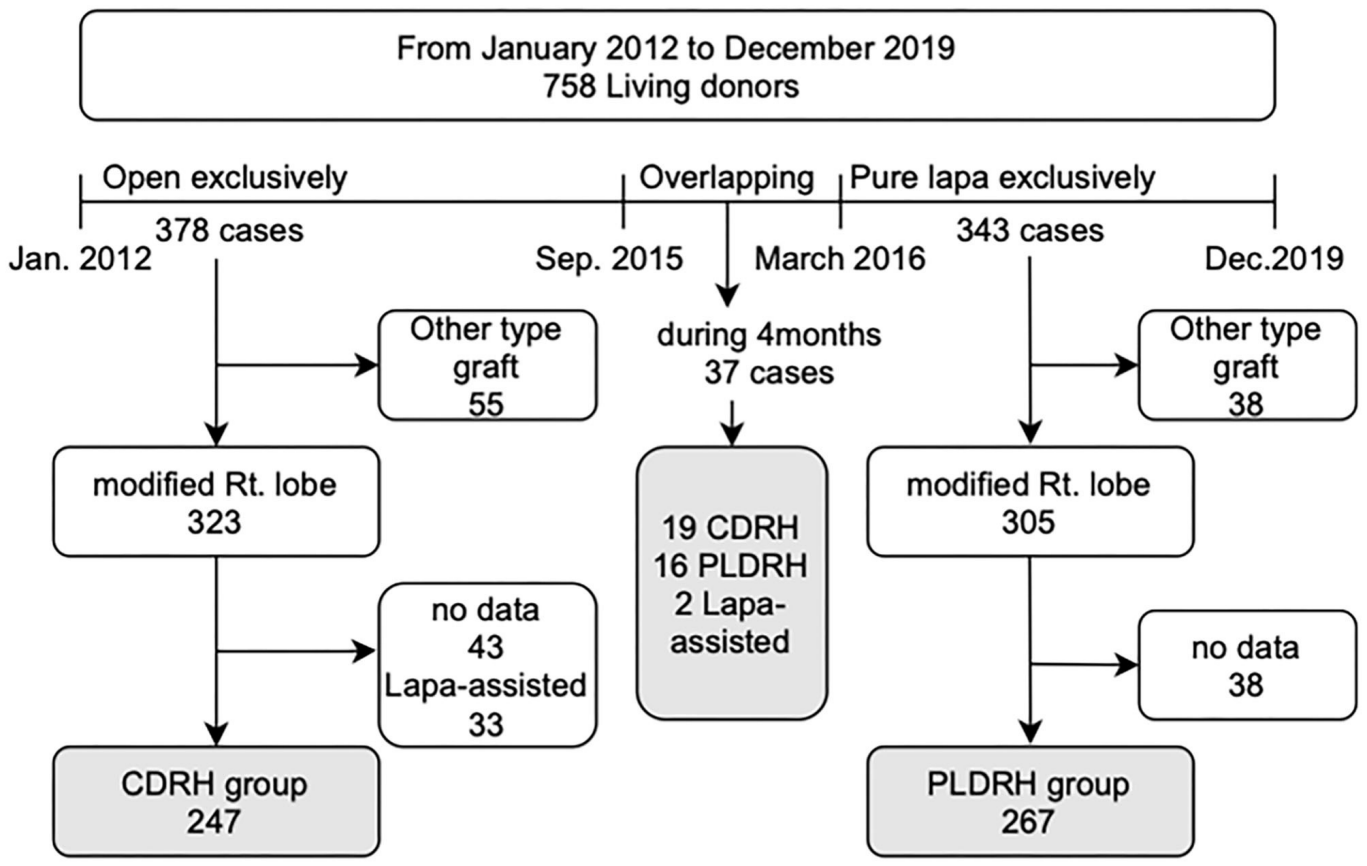
Study design.

This study was approved by the Institutional Review Board of Seoul National University Hospital (IRB no: H-2101-128-1190). We retrospectively reviewed the medical records of 758 live liver donors who underwent hepatectomy between January 2012 and December 2019 at Seoul National University Hospital. The study design is illustrated in Figure 1. We divided the data into two periods: the first period (January 2012-September 2015) during which almost all donor hepatectomies were performed using the conventional open procedure and the second period (March 2016-December 2019) when PLDH was standardized. We excluded donors who underwent donor hepatectomy between the two periods (between October 2015 and February 2016). We also excluded donors who underwent hepatectomy other than right hepatectomy (extended right lobe, left lobe, and left lateral section), those who underwent laparoscopy-assisted donor right hepatectomy, and those whose bench-surgery time was not recorded.

### Bench Procedure and Subgroup Classification

**Figure 2:**
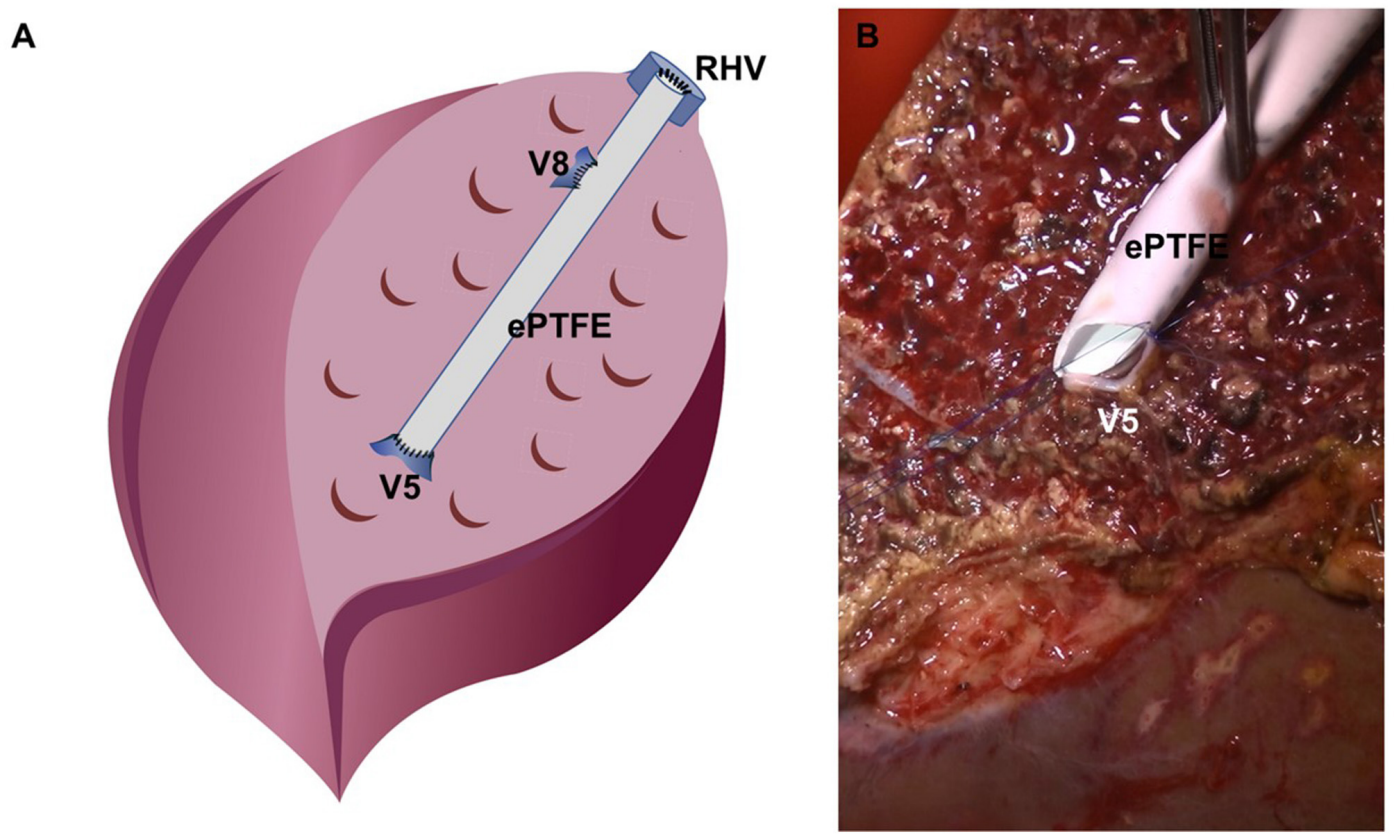
Reconstruction for the middle hepatic vein branch at the bench. **(A)** Schematic figure of bench work, **(B)** surgical field image of V5 darinage using the ePTFE graft.

The bench procedure used for LDLT at our center using a right liver graft has been previously described in detail (11, 12). On graft removal, cold perfusion is initiated using the histidine-tryptophan-ketoglutarate (HTK) solution administered *via* the right portal vein. Second, the temporary clip of the middle hepatic vein (MHV) branch and right inferior hepatic vein (RIHV) is removed to ensure adequate drainage of the anterior section. Subsequently, the hepatic artery and bile duct are washed with heparin-containing HTK solution from a pre-filled syringe. Finally, the donor and bench surgeon decide on the type of reconstruction for the MHV branch. Here, synthetic vascular grafts of thin-walled expanded polytetrafluoroethylene (ePTFE) with 6- or 7-mm internal diameter were used (Figure 2).

Anatomical variations of the graft vascular network may affect bench-surgery time. Therefore, the following factors were considered: (a) portal vein variation with two openings in the graft that required direct venoplasty or Y-graft reconstruction using the recipient's portal vein; (b) more than two openings for V5 or V8 requiring complex reconstruction of the MHV using ePTFE, and (c) presence of a sizable RIHV requiring venoplasty for easy anastomosis later.

### Bench-Surgery Time

After liver retrieval, the right portal vein was declamped on the back table, and perfusion using cold HTK solution was initiated. The bench operation time was defined as the time from the initiation of HTK perfusion to completion of venous reconstruction of the MHV branches as well as dissection and trimming of the portal vein, hepatic artery, and bile duct. Reconstruction of the portal vein and hepatic vein was considered a factor that may affect the time of bench surgery; therefore, vascular anatomical variations were taken into consideration during subgroup analysis.

### Statistical Analysis

Results of descriptive analysis of quantitative data is expressed as mean ± standard deviation. Categorical variables are presented as numbers and percentages. Continuous variables were compared using Student's *t*-test, and categorical variables were compared using the chi-square test or Fisher's exact test, as appropriate. Statistical significance was set at *P*-value of < 0.05. All statistical analyses were performed using the SPSS software (version 23; SPSS Inc., Chicago, IL, USA).

## Results

A total of 247 donors underwent CDRH during the first period and 267 underwent PLDRH during the second period. During the overlapping period between the first and second period, 37 donor hepatectomies, including 19 cases of CDRH, 16 cases of PLDRH, and 2 cases of LDRH, were performed.

The perioperative data of the donors are presented in Table 1. There were no significant differences between the two groups, except less estimated blood loss (331.4 vs. 239.2 min; *P* < 0.001) and shorter length of hospital stay (8.4 vs. 7.2 days; *P* < 0.001) in the PLDRH group than in the CDRH group.

**Table 1:** Demographic characteristics of donors, liver grafts, and recipients.

	**CDRH group** **(***n*** = 247)**	**PLDRH group** **(***n*** = 267)**	***P*** **-value**
**Donor-related variables**			
Age, mean ± SD, years	34.9 ± 11.9	33.2 ± 10.8	0.084
Sex, *n* (%)			0.057
Male	160 (64.8)	151 (56.6)	
Female	87 (35.2)	116 (43.4)	
BMI, mean ± SD, kg/m^2^	23.3 ± 3.3	23.7 ± 3.3	0.245
Estimated blood loss, mean ± SD, ml	331.4 ± 177.0	239.2 ± 197.0	<0.001
Intraoperative transfusion, *n* (%)	0	0	Not significant
Length of hospital stay, mean ± SD, days	8.4 ± 1.5	7.2 ± 2.1	<0.001
**Graft-related variables**			
Bench-surgery time, mean ± SD, min	39.5 ± 17.5	49.3 ± 19.9	<0.001
Actual graft weight, mean ± SD, mg	732.9 ± 153.5	716.7 ± 140.1	0.118
**Recipient-related variables**			
Age, mean ± SD, years	53.4 ± 10.1	55.2 ± 10.7	0.048
Sex, *n* (%)			0.645
Male	174 (70.4)	193 (72.3)	
Female	73 (29.6)	74 (27.7)	
Liver etiology, *n* (%)			0.010
HBV	156 (63.2)	151 (56.6)	
HCV	24 (9.7)	13 (4.9)	
Alcoholic	32 (13.0)	58 (21.7)	
Others	35 (14.1)	45 (16.8)	
Hepatocellular carcinoma, *n* (%)	147 (59.5)	163 (61.0)	0.722
MELD, mean ± SD	15.1 ± 6.8	14.5 ± 6.2	0.312
GRWR, mean ± SD, %	1.29 ± 0.3	1.21 ± 0.3	0.001
ABO incompatibility, *n* (%)	15 (6.1)	50 (18.7)	<0.001

*CDRH, conventional donor right hepatectomy; PLDRH, pure laparoscopic donor right hepatectomy; SD, standard deviation; BMI, body mass index; HBV, hepatitis B virus; HCV, hepatitis C virus; MELD, Model for End-Stage Liver Disease; GRWR, graft-to-recipient weight ratio*.

The proportion of participants with vascular variation who required additional procedures, including portal vein variation with two openings (*P* = 0.670), more than two openings of V5 or V8 (*P* = 0.856), and sizable RIHV (*P* = 0.969), was similar between the CDRH and PLDRH groups (Table 2).

**Table 2:** Proportion of participants with vascular variation related to the graft in the CDRH and PLDRH groups.

	**CDRH group** ***N*** **= 247**	**PLDRH group** ***N*** **= 267**	***P*** **-value**
Vasculature variation at the graft, *n* (%)			0.969
Conventional	165 (67)	183 (68)	0.674
Multiple portal vein openings	22 (9)	21 (8)	0.670
More than two sizeable MHV branches	45 (18)	47 (18)	0.856
Requiring RIHV venoplasty	15 (6)	16 (6)	0.969

*Values are stated as n (%)*.

*CDRH, conventional donor right hepatectomy; PLDRH, pure laparoscopic donor right hepatectomy; MHV, middle hepatic vein; RIHV, right inferior hepatic vein*.

The mean bench-surgery time was longer in the PLDRH group than in the CDRH group (49.3 vs. 39.5 min; *P* < 0.001). We performed a subgroup analysis excluding factors affecting the bench-surgery time in both groups. First, subgroup analysis was performed by excluding patients who underwent direct venoplasty or Y-graft patch reconstruction of the two portal vein openings. Second, another subgroup analysis was performed by excluding patients who underwent reconstruction with more than two openings of MHV tributaries or conversely no reconstruction of MHV tributaries. The last subgroup analysis was performed by excluding patients who underwent venoplasty for RIHV(s) using back-table procedures. In all three subgroup analyses, the mean bench-surgery time was longer in the PLDRH group than in the CDRH group (47.4 vs. 38.4 min; *P* < 0.001, 48.7 vs. 37.4 min; *P* < 0.001, 48.3 vs. 36.6 min; *P* < 0.001, respectively) (Table 3).

**Table 3:** Subgroup analysis based on variations in vascular of liver grafts.

**Bench time, mean ± SD, min**	**CDRH group**	**PLDRH group**	***P*** **-value**
Excluding type II and III portal vein variation	(*n* = 225)	(*n* = 246)	<0.001
	38.4 ± 16.7	47.4 ± 17.9	
Excluding >2 openings of MHV tributaries	(*n* = 180)	(*n* = 199)	<0.001
	37.4 ± 13.2	48.7 ± 14.8	
Excluding RIHV venoplasty	(*n* = 165)	(*n* = 183)	<0.001
	36.6 ± 12.4	48.3 ± 14.0	

*Values are stated as mean ± standard deviation*.

*CDRH, conventional donor right hepatectomy; PLDRH, pure laparoscopic donor right hepatectomy; MHV; middle hepatic vein; RIHV, right inferior hepatic vein*.

## Discussion

Since the first application of donor hepatectomy using the laparoscopic method and its reporting in 2002 (2), PLDH is now widely performed (6, 9). Our center reported its safety and feasibility by the experience of >300 cases of PLDH, mainly right hepatectomy, from 2016 to 2019 (7). In addition, an effective surgical technique has been suggested for various anatomical variations in PLDRH after overcoming the learning curve (13–15). However, additional studies on the recipient's biliary complications and long-term outcomes are required (6, 7).

The quality of the graft in LDLT is vital. In the case of PLDRH, the operation time, time to liver graft removal, and warm ischemia time has been reported to be relatively longer than those of CDRH (7). In particular, prolonged warm ischemia time is believed to be related to various complications; therefore, long-term outcomes need to be analyzed (16). Bench-surgery time is an important factor impacting graft quality, as it is related to cold ischemia time (17–19). The graft quality, such as if additional trimming or bench-surgery procedures are required, can affect bench-surgery time. A factor affecting the bench-surgery time is related to the vascular reconstruction of the graft, such as portal vein variation (types II and III), MHV tributary reconstruction, and RIHV venoplasty. In this study, we compared the time taken for the bench-surgery time of the CDRH and PLDRH groups after liver graft removal. We conducted a subgroup analysis for factors that may affect the bench-surgery time in PLDRH.

We predicted that the vascular stump was relatively short and complex in the PLDRH group, so it was expected to impact the bench-surgery time. As described in our previous reports, while cutting and suturing of the remnant donor side was performed in the CDRH group (11, 20–22), vascular divisions were performed using a vascular stapler in the PLDRH group (4, 5, 13, 15, 23). Additionally, during PLDRH, the right portal vein was transected with a bilateral stapler, and the right hepatic vein was divided with a unilateral linear stapler. These metallic materials occupy space; therefore, the stump of the vascular network inevitably became shorter than that in the CDRH group. This is a factor that may present challenges during recipient reconstruction; therefore, additional manipulation such as trimming the stump or dissection of the surrounding liver parenchyma may be required. When dividing the bile duct, we clamped the double metal clips on the remnant side of the bile duct and cut the graft side of the bile duct. Remnant Glisson's tissue, including the hilar plate, was divided using a Hem-O-Lok clip or an endostapler. In particular, because the Hem-O-Lok clip occupies space and hinders bile duct reconstruction, it is often removed during bench surgery, and suture closure of the caudate branches is performed again. These additional manipulations on the vascular stumps and bile duct divisions may have contributed to the increase in the bench-surgery time.

The proportion of vascular variations that required further procedures, including portal vein variation with two openings, more than two openings of the MHV tributaries, and sizable RIHV, was similar between the CDRH and PLDRH groups because there have been no absolute contraindications for PLDRH since March 2016. Although there was no difference in vascular variation between the two groups, additional procedures may be required to facilitate reconstruction by trimming, attaching fence, and direct venoplasty for relatively short vascular stumps. Taken together, it can be concluded that the additional procedure required for trimming short vascular stumps, hepatic artery, and remnant Glisson's tissues other than vascular variation might have affected the bench-surgery time in the PLDRH group compared with that in the CDRH group. This reflects the importance of the skill of not only donor surgeons but also bench and recipient operators to avoid PLDRH adverse effects on recipient outcomes. In other words, a potential limitation that can arise from a shorter stump in the PLDRH group than in the CDRH group must be addressed during bench surgery.

This study has some limitations. First, this was a single-center retrospective study, with a relatively small sample size. Second, the learning curve of the bench surgery operators was not accurately considered. However, the surgeon factor was considered to have been minimized because surgery was performed under the proctorship of Seoul National University Hospital Liver Transplant Team, who not only possess considerable experience in laparoscopic hepatic resection but also in LDLT.

In conclusion, the bench-surgery time was longer in the PLDRH group regardless of whether the vascular network was reconstructed. Not only is expertise in donor surgery important but also competence and experience in bench surgery and recipient surgery are crucial for PLDRH to be safe and feasible.

## Data Availability Statement

The raw data supporting the conclusions of this article will be made available by the authors, without undue reservation.

## Ethics Statement

This study was approved by the Institutional Review Board of Seoul National University Hospital (IRB no: H-2101-128-1190). Written informed consent for participation was not required for this study in accordance with the national legislation and the institutional requirements.

## Author Contributions

KH and SKH made the concept and design. KH, SKH, ESH, SS, and SyH acquired, analyzed, and interpreted the data. KH and SKH drafted the manuscript. KH, SKH, ESH, SS, SyH, J-ML, YC, N-JY, K-WL, and K-SS critically revised the manuscript for important intellectual content. All authors contributed to the article and approved the submitted version.

## Conflict of Interest

The authors declare that the research was conducted in the absence of any commercial or financial relationships that could be construed as a potential conflict of interest.

## Publisher's Note

All claims expressed in this article are solely those of the authors and do not necessarily represent those of their affiliated organizations, or those of the publisher, the editors and the reviewers. Any product that may be evaluated in this article, or claim that may be made by its manufacturer, is not guaranteed or endorsed by the publisher.

## References

[1] Chan SC Fan ST. Historical perspective of living donor liver transplantation. World J Gastroenterol. (2008) 14:15–21. 10.3748/wjg.14.15 18176956 PMC2673383

[2] Cherqui D Soubrane O Husson E Barshasz E Vignaux O Ghimouz M. Laparoscopic living donor hepatectomy for liver transplantation in children. Lancet. (2002) 359:392–6. 10.1016/S0140-6736(02)07598-0 11844509

[3] Hong SK Lee KW Choi Y Kim HS Ahn SW Yoon KC. Initial experience with purely laparoscopic living-donor right hepatectomy. Br J Surg. (2018) 105:751–9. 10.1002/bjs.10777 29579333

[4] Suh KS Hong SK Lee KW Yi NJ Kim HS Ahn SW. Pure laparoscopic living donor hepatectomy: focus on 55 donors undergoing right hepatectomy. Am J Transplant. (2018) 18:434–43. 10.1111/ajt.14455 28787763

[5] Lee KW Hong SK Suh KS Kim HS Ahn SW Yoon KC. One hundred fifteen cases of pure laparoscopic living donor right hepatectomy at a single center. Transplantation. (2018) 102:1878–84. 10.1097/TP.0000000000002229 29684001

[6] Cho HD Samstein B Chaundry S Kim KH. Minimally invasive donor hepatectomy, systemic review. Int J Surg. (2020) 82S:187–91. 10.1016/j.ijsu.2020.06.023 32615320

[7] Hong SK Tan MY Worakitti L Lee JM Cho JH Yi NJ. Pure laparoscopic versus open right hepatectomy in live liver donors: a propensity score-matched analysis. Ann Surg. (2020). [Epub ahead of print]. 10.1097/SLA.0000000000003914 32324692

[8] Hong SK Choi GS Han J Cho HD Kim JM Han YS. Pure laparoscopic donor hepatectomy: a multicenter experience. Liver Transpl. (2021) 27:67–76. 10.1002/lt.25848 32679612

[9] Cherqui D Ciria R Kwon CHD Kim KH Broering D Wakabayashi G. Expert consensus guidelines on minimally invasive donor hepatectomy for living donor liver transplantation from innovation to implementation: a joint initiative from the International Laparoscopic Liver Society (ILLS) and the Asian-Pacific Hepato-Pancreato-Biliary Association (A-PHPBA). Ann Surg. (2021) 273:96–108. 10.1097/SLA.0000000000004475 33332874

[10] Lee JM Shehta A Lee KW Hong SK Cho JH Yi NJ. Donor wound satisfaction after living-donor liver transplantation in the era of pure laparoscopic donor hepatectomy. Surg Endosc. (2021) 35:2265–72. 10.1007/s00464-020-07640-2 32430524

[11] Yi NJ Suh KS Lee HW Cho EH Shin WY Cho JY. An artificial vascular graft is a useful interpositional material for drainage of the right anterior section in living donor liver transplantation. Liver Transpl. (2007) 13:1159–67. 10.1002/lt.21213 17663413

[12] Hong SK Yi NJ Cho JH Lee JM Hong K Han ES. Parietal peritoneum as a novel substitute for middle hepatic vein reconstruction during living donor liver transplantation. Transplantation. (2021) 105:1291–6. 10.1097/TP.0000000000003349 32568956

[13] Park K Shehta A Lee JM Hong SK Yoon KC Cho JH. Pure 3D laparoscopy versus open right hemihepatectomy in a donor with type II and III portal vein variations. Ann Hepatobiliary Pancreatic Surg. (2019) 23:313–8. 10.14701/ahbps.2019.23.4.313 31824995 PMC6893046

[14] Shehta A Lee JM Lee KW Hong SK Cho JH Yi NJ. Pure laparoscopic living donor hepatectomy for donors with right portal vein anatomical variations. Liver Transpl. (2019) 25:1445–54. 10.1002/lt.25582 31169982

[15] Hong SK Suh KS Lee JM Cho JH Yi NJ Lee KW. New technique for management of separate right posterior and anterior portal veins in pure 3d laparoscopic living donor right hepatectomy. J Gastrointest Surg. (2020) 24:462–3. 10.1007/s11605-019-04350-6 31485905

[16] Olthof PB van Golen RF Meijer B van Beek AA Bennink RJ Verheij J. Warm ischemia time-dependent variation in liver damage, inflammation, and function in hepatic ischemia/reperfusion injury. Biochim Biophys Acta Mol Basis Dis. (2017) 1863:375–85. 10.1016/j.bbadis.2016.10.022 27989959

[17] Pan ET Yoeli D Galvan NTN Kueht ML Cotton RT O'Mahony CA. Cold ischemia time is an important risk factor for post–liver transplant prolonged length of stay. Liver Transpl. (2018) 24:762–8. 10.1002/lt.25040 29476693

[18] Scotté M Dousset B Calmus Y Conti F Houssin D Chapuis Y. The influence of cold ischemia time on biliary complications following liver transplantation. J Hepatol. (1994) 21:340–6. 10.1016/S0168-8278(05)80311-3 7836702

[19] Rodrigues MG Castro PMV Almeida TC Danziere FR Sergi Filho FA Zeballos Sempertegui BE. Impact of cold ischemia time on the function of liver grafts preserved with Custodiol. Transplant Proc. (2021) 53:661–4. 10.1016/j.transproceed.2020.03.002 33139037

[20] Yi NJ Suh KS Cho JY Lee HW Cho EH Yang SH. Three-quarters of right liver donors experienced postoperative complications. Liver Transpl. (2007) 13:797–806. 10.1002/lt.21030 17539000

[21] Yi NJ Suh KS Suh SW Chang YR Hong G Yoo T. Excellent outcome in 238 consecutive living donor liver transplantations using the right liver graft in a large volume single center. World J Surg. (2013) 37:1419–29. 10.1007/s00268-013-1976-y 23467924

[22] Suh KS Suh SW Lee JM Choi Y Yi NJ Lee KW. Recent advancements in and views on the donor operation in living donor liver transplantation: a single-center study of 886 patients over 13 years. Liver Transpl. (2015) 21:329–38. 10.1002/lt.24061 25488794

[23] Hong SK Lee KW Kim HS Yoon KC Ahn SW Choi JY. Optimal bile duct division using real-time indocyanine green near-infrared fluorescence cholangiography during laparoscopic donor hepatectomy. Liver Transpl. (2017) 23:847–52. 10.1002/lt.24686 27935193

